# Evaluation of the Comparability of Wantai Wan200+ Instrument with Routine Laboratory Assays for 21 Different Analytes

**DOI:** 10.3390/jcm13082246

**Published:** 2024-04-12

**Authors:** Ilaria Talli, Andrea Padoan, Chiara Cosma, Giulia Furlan, Martina Zaninotto, Lucio Marchioro, Paola Galozzi, Daniela Basso, Mario Plebani

**Affiliations:** 1 Department of Medicine (DIMED), University of Padova, 35128 Padova, Italy; ilaria.talli@studenti.unipd.it (I.T.); andrea.padoan@unipd.it (A.P.); chiara.cosma@unipd.it (C.C.); paola.galozzi@unipd.it (P.G.); daniela.basso@unipd.it (D.B.); 2 Laboratory Medicine Unit, University Hospital of Padova, 35128 Padova, Italy; 3 QI.LAB.MED, Spin-Off of the University of Padova, 35011 Campodarsego, Italy; giulia.furlan.5@studenti.unipd.it (G.F.); martina.zaninotto@aopd.veneto.it (M.Z.); luma53@libero.it (L.M.)

**Keywords:** Wantai Wan200+, laboratory automation, fully automated analyzer, clinical biochemistry analyzer, immunometry analyzer

## Abstract

**Background**: We compared the performance of 21 different assays performed by the Wantai Wan200+ (Wantai BioPharm, Beijing, China) with respect to other methods in use at the University Hospital of Padova (AOPD), Italy. **Methods**: The plasma (P) or serum (S) of 5027 leftover samples, collected from May to Sept 2023, was either analyzed or frozen at −20 °C. Beckman DXI800 (DXI), Roche Cobas 8000 e801 (RC), Snibe Maglumi 4000 plus (SM), DiaSorin Liaison XL (DL) and Binding Site Optilite (BS) equipment were used at the AOPD. P-procalcitonin (PCT), DXI; P-Troponin I (TnI), DXI; S-CA125, DXI; S-free PSA (f-PSA), DXI; S-total PSA (t-PSA), DXI; S-IL6, SM; P-Troponin T (TnT), RC; P-NT-proBNP, RC; P-Neuron-Specific Enolase (NSE), RC; S-CA15-3, DL; S-CA19-9, DL; S-AFP, DL; and S-CEA, DL were tested in fresh samples. P-Myoglobin (Myo), DXI; P-Cyfra21-1, RC; S-β2 microglobulin (B2MIC), BS; S-HE4, SM; S-PGI, SM; S-PGII, SM; S-CA72-4, SM; and S-CA50, SM were analyzed in frozen and thawed samples. Bland–Altman (BA), Passing–Bablok (PB) and Cohen’s Kappa (CKa) metrics were used as statistics. **Results**: An excellent comparability profile was found for 11 analytes. For example, the t-PSA CKa was 0.94 (95%CI: 0.90 to 0.98), and the PB slope and intercept were 1.02 (95%CI: 0.99 to 1.03) and 0.02 (95%CI: 0.01 to 0.03), respectively; the BA bias was 2.25 (95%CI: −0.43 to 4.93). Ten tested measurands demonstrated a suboptimal comparability profile. Biological variation in EFLM (EuBIVAS) performance specifications was evaluated to assess the clinical relevance of measured biases. **Conclusions**: Evaluation of the Wantai Wan200+’s performance suggests that between-method differences did not exceed the calculated bias. Metrological traceability may influence the comparisons obtained for some measurands.

## 1. Introduction

The term automation generally refers to the substitution of manual efforts and intelligence with mechanical, electrical or computerized processes [1]. In the contemporary setting of laboratory medicine, the adoption of systems that automate most of the manual phases of routine activities has significantly improved different tasks performed during the total testing process (TTP), leading to advancements in the overall laboratory performance [2]. In this scenario, “quality” encompasses a broad concept, and includes analytical quality, labor savings, turnaround time (TAT) [3], safety improvements and result comparability over time and across different locations [4]. In addition, the development of novel technologies that can perform a variety of analyses through different methods may be of significant interest, especially in the process of the reorganization of laboratories, from a compartmentalized to more decision-making-oriented organization that enables the monitoring of each phase of the TTP [3]. To achieve the optimal benefits of an automated system, the instruments should enable both fast and accurate results [3,5]. Instruments able to perform panels of several analytes of both traditional clinical chemistry and immunoassays make the diagnostic process more efficient, with complete traceability of the entire process. The integration of different analytical methods in one single automated platform (TLA) enables the measurement of several physio-pathological parameters for one patient’s sample on the one hand [6]; on the other hand, it reduces the impact of blood collection and the number of samples needed per patient. It is an advantage not only for small laboratories but also for networks of clinical laboratories. In the first case, small laboratories need only one (or a few) instrument(s) to assure the results for a large panel of analytes. In the second situation, it enables the creation of functional backups through the overlapping of the same diagnostic technology [7] and reduces costs, improving data comparability. The development of novel multi-analyte platforms, together with technological advancements, is considered important for in vitro diagnostic manufacturers. Moreover, the comparison of these novel instruments with already validated technologies represents an important task to guarantee the accuracy and reliability of the test results for clinical diagnostic purposes [8].

Since laboratory results are essential for clinical decision making, it is important to achieve true and precise measurements using standardization and metrological approaches, employing common measurement units and traceability of measured values [9]. This may entail a lengthy development pathway for newly discovered biomarkers; however, among the established commercially available measurands, only a small number of them present certified reference materials (CRMs) or reference measurement procedures (RMPs). This latter fact represents a major concern, alongside the lack of standardization in pre- and post-analytical phases, which affects the interpretability of results between different IVD-MDs used by various laboratories [9,10].

In this regard, the new Wantai Wan200+ (Wantai BioPharm, No. 31 Kexueyuan Road, Changping District, Beijing, China), a high-throughput instrument for the evaluation of a large panel of different analytes, from clinical chemistry to immunometric assays, has the potential to be a valuable choice in many settings and circumstances.

This study aimed to compare the analytical performance of Wantai Wan200+ concerning a large series of 21 measurands, which ranged from markers of inflammatory disorders to tumors and cardiovascular diseases with respect to the methods routinely used in the Laboratory Medicine Unit at the University Hospital of Padova (AOPD). Furthermore, the metrological traceability of evaluated measurands was studied to investigate the potential causes of a limited between-assay comparability.

## 2. Materials and Methods

### 2.1. Sample Collection and Preparation

To evaluate the performance of the Wantai instrument (Wan200+) (Wantai BioPharm, No. 31 Kexueyuan Road, Changping District, Beijing, China), a total of 5027 leftover routine samples from the Laboratory Medicine Unit of the University Hospital of Padova (the period from 15 May to 11 September) were analyzed. Samples were collected, including from both healthy subjects and patients with different pathological disorders. To collect samples for a wide range of measurements, for each analyte, a ratio of 1:1 or 2:1 (rate of samples with normal values vs. samples with pathological values based on reference intervals) was used during sample collection. Samples were centrifuged at 3500× *g* 5 min, and serum or plasma was transferred into a second tube. To safeguard the confidentially of patients’ information, each sample was decanted into a test tube (Tecan, Röhren, Sarstedt, Germany, REF. 55475030), which was then deidentified and labeled with a unique and progressive identifier number.

### 2.2. Sample analysis

Samples were immediately analyzed after collection with methods routinely used at the Laboratory Medicine Unit at the University Hospital of Padova, as follows:

1. Plasma procalcitonin (PCT) (*n* = 300 samples), plasma Troponin I (TROPI) (*n* = 398 samples), serum CA 125 (*n* = 300 samples), serum-free PSA (f-PSA) (*n* = 300 samples) and serum total PSA (t-PSA) (*n* = 356 samples) samples were analyzed using a DXI 800 (Beckman Coulter, Brea, CA, USA);
2. Serum IL-6 (*n* = 300 samples) samples were analyzed using a Snibe Diagnostic (Shenzen, China) Maglumi 4000 plus;
3. Plasma troponin T (TROPT) (*n* = 317 samples), plasma NT-proBNP (*n* = 301 samples) and plasma Neuron-Specific Enolase (NSE) (*n* = 126 samples) samples were analyzed using a Roche (Basel, Switzerland) Cobas 8000 e801;
4. Serum CA 15-3 (*n* = 300 samples), serum CA 19-9 (*n* = 302 samples), serum AFP (*n* = 305 samples) and serum CEA (*n* = 300 samples) samples were analyzed using a DiaSorin (Sallugia, Italy) Liaison XL.

For each of the previously reported analytes, more than 300 samples were collected, except for NSE samples (120 samples).

Indeed, for each of the following analytes, at least 120 samples were collected, except for β-2 microglobulin (B2MIC) samples, of which there were more than 200, and CA72-4, of which there were 108.

1. Plasma Myoglobin (*n* = 127 samples) samples were analyzed using a Beckman Coulter (Brea, CA, USA) DXI 800;
2. Plasma Cyfra 21-1 (Cyfra) (*n* = 129 samples) samples were analyzed using a Roche (Basel, Switzerland) Cobas 8000 e801;
3. Serum β-2 microglobulin (B2MIC) (*n* = 206 samples) samples were analyzed using a Binding Site (Birmingham, Great Britain), Optilite;
4. Serum HE4 (*n* = 123 samples), serum PGI (*n* = 164 samples), serum PGII (*n* = 164 samples), serum CA 72-4 (*n* = 108 samples) and serum CA 50 (*n* = 120 samples) samples were analyzed using a Snibe Diagnostic (Shenzen, China) Maglumi 4000 plus.

Afterwards, some measurands were analyzed using the Wantai Wan200+ within 24 h after collection (freshly collected samples) while, for other measurands, particularly those that are less frequently requested in routine analyses, aliquots of plasma or serum were frozen before analysis (frozen samples). Frozen samples were kept at −20 °C for no more than three months until analysis using the Wantai Wan200+; after thawing, specimens were centrifuged at 3500× *g* 5 min before performing Wan200+ analyses.

More precisely, the list of measurands analyzed in fresh or frozen samples is as follows:

- Freshly collected samples: plasma procalcitonin (PCT), plasma Troponin I (TROPI), serum CA 125, serum-free PSA (f-PSA), serum total PSA (t-PSA), serum IL-6, plasma troponin T (TROPT), plasma NT-proBNP, plasma Neuron-Specific Enolase (NSE), serum CA 15-3, serum CA 19-9, serum AFP and serum CEA samples;
- Frozen samples: plasma Myoglobin, plasma Cyfra 21-1 (Cyfra), serum β-2 microglobulin (B2MIC), serum HE4, serum PGI, serum PGII, serum CA 72-4 and serum CA 50 samples.

According to the manufacturers’ instructions, freezing (up to three months) and thawing processes do not impact the analytical results. We evaluated the presence of different results for a number of analytes per analysis, and no statistically significant differences were found.

### 2.3. Wantai Wan200+ Analyses

The Wantai Wan200+ is a high-throughput laboratory instrument that enables the determination of a panel of analytes, from clinical biochemistry to immunometry. Several assays can be performed using one single aliquot for each patient, lowering the number of requested samples per patient and the time to obtain results.

Before starting the measurements, the Wan200+ instrument was calibrated for each analyte, and then at least six repetitions of quality controls (QCs) at different levels (Q1 = negative value; Q2 = pathologic value; Q3 = high pathologic value; Bio-Rad, Hercules, CA, USA) were run. For each QC level, the mean value, the standard deviation (SD) and the variation coefficient (CV) were obtained and used to calculate QC limits (QC mean ± 3 standard deviation) in order to keep the system controlled. Every day, quality controls were run before the analytical session to check they were inside the QC limits (QC mean ± 3 SD). In the case that QC values were outside the acceptable range, either QC, calibrators or both were run again. In the case of a great number of samples in one analytical session, controls were repeated twice or three times during the evaluation.

### 2.4. Statistical Analyses

The mean, standard deviation, median and interquartile ranges were used as descriptive statistics. A Bland–Altman plot was used to evaluate the presence of bias (in percentages). Passing–Bablok non-parametric regression was used to underline whether proportional bias exists between the compared methods. Linearity assumption was checked after any Passing–Bablok regression via a cusum test.

## 3. Results

A comparison between the 21 different assays performed using the Wantai Wan200+ and with the routine assays in use at AOPD was performed both from a qualitative and quantitative point of view. Using internal quality control material that was analyzed daily, the precision of each assay was evaluated, resulting in a range from 0.48% (TROPT Q2) to 7.92% (tPSA Q1).

Of the 21 measured analytes, 11 granted results with a significant agreement with the methods used in routine at AOPD, both for qualitative and quantitative statistical analyses. More specifically, they were IL6, NSE, Myoglobin, HE4, B2MIC, PGII, t-PSA, CA 50, TROPT, CA 15-3 and TROPI (Table 1).

**Figure 1:**
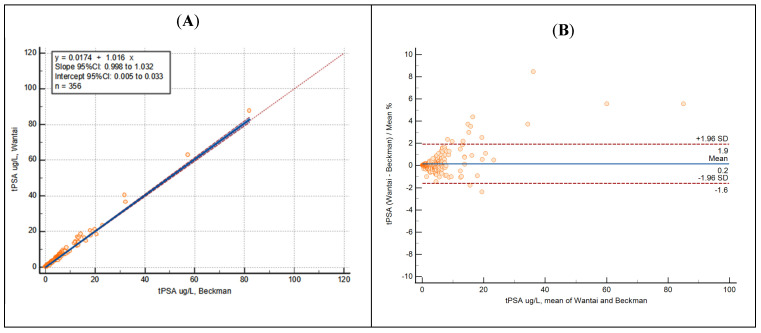
Passing–Bablok regression (**A**) and Bland–Altman analysis (**B**) of t-PSA showing results obtained using Beckman (AOPD) and Wantai Wan200+ equipment.

As an example, Cohen’s Kappa of t-PSA demonstrated a perfect dichotomic agreement, Passing–Bablok regression coefficients showed a perfect method overlap and Bland–Altman analysis underlined the absence of bias (Figure 1).

Statistical analyses for the other 10 measurands demonstrated a suboptimal comparability with respect to the comparator method (Table 2).

**Figure 2:**
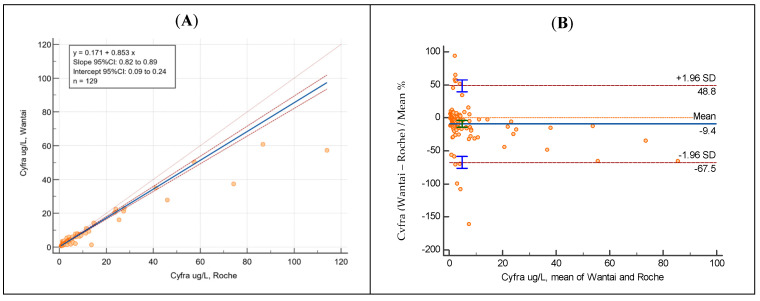
Passing–Bablok regression (**A**) and Bland–Altman analysis (**B**) of Cyfra 21-1 showing results obtained with Roche (AOPD) and Wantai Wan200+ equipment.

As an illustrative example, Passing–Bablok regression coefficients and the Bland–Altman analysis of Cyfra 21-1 are reported below (Figure 2).

## 4. Discussion

In order to guarantee that patients can take advantage of the maximum benefits and the optimal possible treatment using clinical laboratory information, technological advancements in routine laboratory practice are highly recommended. This entails enhancing both the efficiency of laboratory testing and the consistency of results to yield robust and actionable information useful for clinical diagnosis [24].

**Figure 3:**
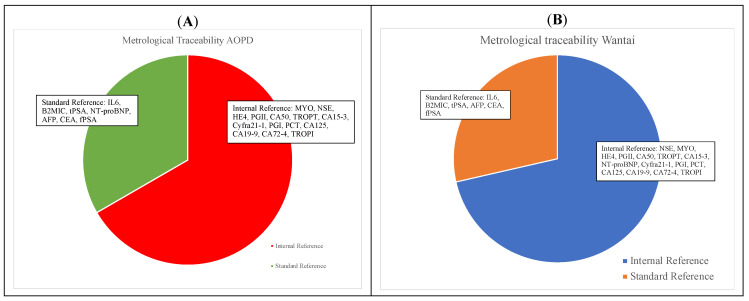
Metrological traceability of comparative AOPD (**A**) and Wantai (**B**) assays.

The Wantai Wan200+ is a highly automated immunoassay, with an interesting throughput, that allows for the provision and release of results for a large series of measurands, including tests for infectious diseases, tumor markers, thyroid, cardiac markers, hormones, inflammatory biomarkers, renal function, diabetes and TORCH, starting from a single serum aliquot. In this study, the comparability of Wantai Wan200+ for 21 measurands was evaluated using a large series of 5027 leftover samples, in comparison with the analytical systems established at the University Hospital of Padova. Among the 21 evaluated measurands, performances varied. A total of 11 analytes demonstrated an excellent comparability profile, both in qualitative and quantitative statistical evaluations, being fully comparable to those obtained using the routine laboratory assays at AOPD. More specifically, these assays were for IL6, NSE, Myoglobin, HE4, β2-microglobulin, PGII, t-PSA, CA 50, Troponin T, CA 15-3 and Troponin I. On the other hand, the remaining 10 tested measurands demonstrated a suboptimal comparability profile (Table 2). Since it is well known that performance characteristics, such as the method comparability, should be objectively compared to well-documented quality specifications [25], we evaluated whether significant differences exceed the analytical performance specifications (APSs). The allowable desirable/minimum bias was calculated based on the biological variation data [26]. Using APS, a limited comparability profile was detected only for NT-proBNP and PGI. Next, we examined the metrological traceability of the Wantai Wan200+ and comparative methods. In fact, it is of utmost importance to ensure that samples for the same patient can be reliably tested in various laboratories (or various instruments within the same laboratory) over time, with each possibly employing a unique analytical assay [27]. The metrological traceability results, reported in Table 3, highlight that some assays of Wantai and routine AOPD refer to different traceability methods: 67% and 71% of performed assays refer to internal reference methods for AOPD and Wantai assays, respectively, while 33% and 29% of performed assays refer to standard reference methods for AOPD and Wantai assays, respectively (Figure 3). It is interesting to note that for IL6, B2MIC, t-PSA, AFP, CEA and f-PSA, the high-order metrological traceability chains are the same both for Wantai assays and comparative assays. For NT-proBNP, a metrological traceability chain was present for the comparator assay, but not for Wantai Wan200+; for PGI, no traceability chains were detected. This fact might partially explain our results, where for NT-proBNP and PGI, a limited comparability was underlined. Indeed, the issue concerning the impact of the absence of CRM and RMP for many measurands on the comparability of test results is well known. It is also widely recognized that standardized test results can enhance the clinical effectiveness and cost-effectiveness of care, thereby reducing the movement of patients across healthcare institutions [9].

It should be noted that for the PGI assay, the original reference population could explain the marked difference in the reference intervals (RIs) with respect to the comparator assay [28]; furthermore, RIs might also vary within different geographical areas [29].

To our knowledge, this is the first study evaluating the comparability of Wantai Wan200+ for a large number of tests. In the literature, different comparability studies have reported the presence of bias across different immunoassays. For example, Fu et al., by assessing the performances of CEA in six immunoassays, found bias values ranging from 1.67% to 13.18% and, hence, underlined the assay-dependent levels of CEA [30]. Walker et al. proposed a bias of 12% in the performance between two immunoassays for assessing PCT in a total of 45 samples; moreover, they found that most of the observed bias occurred starting at a PCT level of 0.5 ng/mL, which is higher than the cut-off for clinical decision for antibiotic therapy [31]. Dipalo et al. assessed the bias of PCT measurements for 100 samples in four automated immunoassays, finding that the mean value is 0.9 ng/mL [32]. Barr et al. analyzed HE4 in 1348 samples in two immunoassays; a mean bias of 16.25% was found between the two immunoassays, where the CLEIA method overestimated HE4 results with respect to the EIA method [33]. Considering the Wantai Wan200+, the findings from this study showed better performances than those reported in the studies mentioned above, demonstrating the value of this analyzer. This study also has some limitations. Firstly, analytical performances were not assessed, although third-part internal quality control measurements were carefully evaluated with respect to the manufacturers’ declared performances. Secondly, for some measurands (e.g., CA 72-4 and CA 50), it was impossible to collect a wide range of values due to the limited amount of tests routinely requested. Additionally, wide confidence intervals were observed for some measurands due to the limited sample size, which could undermine the accuracy of statistical estimates. Furthermore, this study employed different analyzers rather than a single device similar to the Wantai Wan200+.

## 5. Conclusions

In this study, after testing a large number of samples, we verified that the Wantai Wan200+ is able to perform measurements of several different analytes for clinical biochemistry and immunoassay measurands, with a great potential impact in the setting of total laboratory automation (TLA). The possibility of highly automating the Wantai Wan200+ is an advantage in executing repetitive steps and tasks, enhancing both the quality of the results and the overall analytical performance [34,35]. This study underlined that these perspectives were achieved by Wantai Wan200+, even if some limitations based on standardization of measurements still need to be completely addressed to improve the results comparability across platforms.

## Figures and Tables

**Table 1:** Cohen’s Kappa, Passing–Bablok slope and intercept and Bland–Altman bias are reported with their corresponding 95% CI for each analyte.

Analyte	Cohen’s Kappa (95% CI)	Slope (95% CI)	Intercept (95% CI)	Bias (%) (95% CI)	Desirable Bias (%) Estimated by BV *
IL6 [11]	0.846 (95%CI: 0.78 to 0.91)	0.91 (95%CI:0.85 to 0.95)	0.4 (95%CI: 0.15 to 0.55)	−7.01 (95%CI: −14.95 to 0.93) *p* = 0.083	15.61
NSE [12]	0.76 (95%CI: 0.64 to 0.88)	0.91 (95%CI: 0.84 to 0.96)	0.54 (95%CI: −0.46 to 1.97)	−3.01 (95%CI: −5.21 to −0.82) *p* = 0.007	5.76
MYO [13]	0.79 (95%CI: 0.69 to 0.89)	1.02 (95%CI: 0.99 to 1.05)	3.82 (95%CI: 2.02 to 5.19)	7.94 (95%CI: 5.65 to 10.23) *p* < 0.001	12.45
HE4 [14]	0.71 (95%CI: 0.54 to 0.88)	1.00 (95%CI: 0.93 to 1.08)	1.48 (95%CI: −2.90 to 5.28)	3.08 (95%CI: −2.00 to 8.16) *p* = 0.233	4.76
B2MIC [15]	0.63 (95%CI: 0.52 to 0.74)	1.05 (95%CI: 0.93 to 1.18)	−0.19 (95%CI: −0.53 to 0.05)	−0.32 (95%CI: −0.54 to 0.10) *p* = 0.005	2.97
PGII [16]	0.75 (95%CI: 0.57 to 0.93)	1.07 (95%CI: 1.02 to 1.11)	−0.49 (95%CI: −0.98 to 0.004)	0.31 (95%CI: 0.02 to 0.60) *p* = 0.034	9.82
t-PSA [17]	0.94 (95%CI: 0.90 to 0.98)	1.02 (95%CI: 0.998 to 1.03)	0.02 (95%CI: 0.01 to 0.03)	2.25 (95%CI: −0.43 to 4.93) *p* = 0.099	10.64
CA 50	0.66 (95%CI: 0.04 to 1.00)	0.89 (95%CI: 0.85 to 0.93)	0.28 (95%CI: 0.07 to 0.41)	−0.26 (95%CI: −0.58 to 0.07) *p* = 0.12	-
TROPT [18]	0.80 (95%CI: 0.66 to 0.93)	0.98 (95%CI: 0.97 to 1.01)	0.95 (95%CI: 0.93 to 0.97)	−2.43 (95%CI: −3.25 to −1.71) *p* = 0.146	13.41
CA 15-3 [19]	0.90 (95%CI: 0.85 to 0.96)	1.00 (95%CI: 0.97 to 1.02)	−0.63 (95%CI: −1.11 to −0.21)	0.13 (95%CI: −1.96 to 2.22) *p* = 0.902	9.27
TROPI [20]	0.77 (95%CI: 0.71 to 0.83)	1.03 (95%CI: 0.99 to 1.06)	1.06 (95%CI: 1.02 to 1.10)	4.20 (95%CI: 3.01 to 4.24) *p* = 0.087	9.72

* BV = biological variability from EuBIVAS.

**Table 2:** Cohen’s Kappa, Passing–Bablok slope and intercept and Bland–Altman bias are reported with their corresponding 95% CI for each analyte.

Analyte	Cohen’s Kappa (95% CI)	Slope (95% CI)	Intercept (95% CI)	Bias (%) (95% CI)	Desirable/Minimum Bias (%) Estimated by BV *
NT-proBNP [21]	0.93 (95%CI: 0.89 to 0.97)	1.21 (95%CI: 1.20 to 1.22)	−8.18 (95%CI: −9.56 to −7.04)	8.45 (95%CI: 6.74 to 10.16) *p* < 0.0001	4.17/6.26
Cyfra 21-1 [19]	0.79 (95%CI: 0.67 to 0.89)	0.85 (95%CI: 0.82 to 0.89)	0.17 (95%CI: 0.09 to 0.24)	−9.4 (95%CI: −14.6 to −4.21) *p* = 0.001	8.87/13.30
PGI [16]	0.15 (95%CI: −0.002 to 0.31)	0.83 (95%CI: 0.78 to 0.87)	0.37 (95%CI: −0.87 to 3.13)	−17.72 (95%CI: −20.77 to −14.68) *p* < 0.001	6.11/9.16
PCT [22]	0.93 (95%CI: 0.88 to 0.96)	0.76 (95%CI: 0.74 to 0.77)	0.02 (95%CI: 0.02 to 0.03)	−2.17 (95%CI: −3.35 to −0.998) *p* = 0.0003	16.77/25.15
CA 125 [19]	0.67 (95%CI: 0.58 to 0.76)	0.67 (95%CI: 0.65 to 0.69)	3.38 (95%CI: 3.02 to 3.78)	−10.43 (95%CI: −13.62 to −7.23) *p* < 0.001	10.45/15.67
CA 19-9 [19]	0.67 (95%CI: 0.59 to 0.75)	0.77 (95%CI: 0.72 to 0.84)	1.97 (95%CI: 1.52 to 2.60)	−9.97 (95%CI: −15.57 to −4.38) *p* < 0.001	14.04/21.05
AFP [19]	0.89 (95%CI: 0.82 to 0.96)	1.20 (95%CI: 1.18 to 1.23)	−0.56 (95%CI: −0.64 to −0.49)	1.14 (95%CI: 0.63 to 1.66) *p* < 0.001	17.70/26.54
CEA [19]	0.98 (95%CI: 0.96 to 1.00)	1.32 (95%CI: 1.28 to 1.36)	0.26 (95%CI: 0.17 to 0.36)	4.51 (95%CI: −0.05 to 9.07) *p* = 0.053	15.04/22.55
f-PSA [17]	0.12 (95%CI: 0.01 to 0.23)	0.69 (95%CI: 0.98 to 0.70)	0.002 (95%CI: −0.002 to 0.008)	−0.26 (95%CI: −0.30 to −0.23) *p* < 0.0001	11.69/17.53
CA 72-4 [23]	0.79 (95%CI: 0.55 to 1.00)	2.32 (95%CI: 1.70 to 7.17)	−0.63 (95%CI: −2.96 to −0.33)	0.57 (95%CI: 0.29 to 0.85) *p* < 0.0001	28.75/43.12

* BV = biological variability from EuBIVAS.

**Table 3:** The traceability methods for both Wantai and comparative assays for each analyte (Figure 3 shows the percentage of assays with a declared metrological traceability (panel A for comparator methods, AOPD; panel B for Wantai system)). Metrological traceability data were obtained by manufacturers’ inserts.

Analyte	Metrological Traceability of Comparator Assays	Metrological Traceability of Wantai
IL6	NIBSC 89/548	NIBSC 89/548
NSE *	**Enzymun Test NSE**	**Inhouse reference material**
MYO *	Access internal reference material	No high-order traceability
HE4 *	SNIBE internal reference material	Inhouse reference material
B2MIC	1st International Standard NIBSC β2M	First International Standard for Beta2 Microglobulin NIBSC code: B2M
PGII *	SNIBE internal reference material	No high-order traceability
t-PSA	WHO 96/670	WHO 96/670
CA 50 *	SNIBE internal reference material	Inhouse reference material
TROPT *	**Enzymun Test Troponin T (cardiac)**	**Inhouse reference material**
CA 15-3 *	**IRMA CA15-3 (Fujirebio)**	**Inhouse reference material**
TROPI *	Access internal reference material	No high-order traceability
NT-proBNP *	**NT-proBNP (1-76)**	**No high-order traceability**
Cyfra 21-1 *	**Enzymun Test Cyfra21-1**	**Inhouse reference material**
PGI *	SNIBE internal reference material	No high-order traceability
PCT *	Access internal reference material	No high-order traceability
CA 125 *	Access internal reference material	Inhouse reference material
CA 19-9 *	**IRMA CA19-9 (Fujirebio)**	**Inhouse reference material**
AFP	MRC 72/225	First IRP WHO Reference Standard 72/225
CEA	MRC 73/601	First IRP WHO Reference Standard 73/601
f-PSA	WHO 96/668	WHO 96/668
CA 72-4 *	SNIBE internal reference material	Inhouse reference material

* Wantai assays with inhouse reference material or no high-order traceability; in bold are the discordant metrological traceability chains.

## Data Availability

The data presented in this study are available on request from the corresponding author.

## References

[1] Riben M. (2015). Laboratory Automation and Middleware. Surg. Pathol. Clin..

[2] Zaninotto M., Plebani M. (2010). The “hospital central laboratory”: Automation, integration and clinical usefulness. Clin. Chem. Lab. Med..

[3] Dolci A., Giavarina D., Pasqualetti S., Szőke D., Panteghini M. (2017). Total laboratory automation: Do stat tests still matter?. Clin. Biochem..

[4] Plebani M. (2007). Errors in laboratory medicine and patient safety: The road ahead. Clin. Chem. Lab. Med..

[5] Lubin I.M., Astles J.R., Shahangian S., Madison B., Parry R., Schmidt R.L., Rubinstein M.L. (2021). Bringing the clinical laboratory into the strategy to advance diagnostic excellence. Diagnosis.

[6] Lippi G., Da Rin G. (2019). Advantages and limitations of total laboratory automation: A personal overview. Clin. Chem. Lab. Med..

[7] Hawker C.D. (2017). Nonanalytic Laboratory Automation: A Quarter Century of Progress. Clin. Chem..

[8] Nam Y., Lee J.H., Kim S.M., Jun S.H., Song S.H., Lee K., Song J. (2022). Periodic Comparability Verification and Within-Laboratory Harmonization of Clinical Chemistry Laboratory Results at a Large Healthcare Center With Multiple Instruments. Ann. Lab. Med..

[9] Miller W.G., Myers G., Cobbaert C.M., Young I.S., Theodorsson E., Wielgosz R.I., Westwood S., Maniguet S., Gillery P. (2022). Overcoming challenges regarding reference materials and regulations that influence global standardization of medical laboratory testing results. Clin. Chem. Lab. Med..

[10] Panteghini M., Braga F. (2020). Implementation of metrological traceability in laboratory medicine: Where we are and what is missing. Clin. Chem. Lab. Med..

[11] Cava F., González C., Pascual M.J., Navajo J.A., González-Buitrago J.M. (2000). Biological variation of interleukin 6 (IL-6) and soluble interleukin 2 receptor (sIL2R) in serum of healthy individuals. Cytokine.

[12] Carobene A., Guerra E., Locatelli M., Ceriotti F., Sandberg S., Fernandez-Calle P., Coşkun A., Aarsand A.K., European Federation of Clinical Chemistry and Laboratory Medicine Working Group on Biological Variation (2018). Providing Correct Estimates of Biological Variation-Not an Easy Task. The Example of S100-β Protein and Neuron-Specific Enolase. Clin. Chem..

[13] Ross S.M., Fraser C.G. (1998). Biological variation of cardiac markers: Analytical and clinical considerations. Ann. Clin. Biochem..

[14] Braga F., Ferraro S., Mozzi R., Panteghini M. (2014). The importance of individual biology in the clinical use of serum biomarkers for ovarian cancer. Clin. Chem. Lab. Med..

[15] Carobene A., Aarsand A.K., Guerra E., Bartlett W.A., Coşkun A., Díaz-Garzón J., Fernandez-Calle P., Jonker N., Locatelli M., Sandberg S. (2019). European Biological Variation Study (EuBIVAS): Within- and Between-Subject Biological Variation Data for 15 Frequently Measured Proteins. Clin. Chem..

[16] Qi Z., Zhang L., Chen Y., Ma X., Gao X., Du J., Zhang F., Cheng X., Cui W. (2015). Biological variations of seven tumor markers. Clin. Chim. Acta.

[17] Carobene A., Guerra E., Locatelli M., Cucchiara V., Briganti A., Aarsand A.K., Coşkun A., Díaz-Garzón J., Fernandez-Calle P., Røraas T. (2018). Biological variation estimates for prostate specific antigen from the European Biological Variation Study; consequences for diagnosis and monitoring of prostate cancer. Clin. Chim. Acta.

[18] Meijers W.C., van der Velde A.R., Muller Kobold A.C., Dijck-Brouwer J., Wu A.H., Jaffe A., de Boer R.A. (2017). Variability of biomarkers in patients with chronic heart failure and healthy controls. Eur. J. Heart Fail..

[19] Coşkun A., Aarsand A.K., Sandberg S., Guerra E., Locatelli M., Díaz-Garzón J., Fernandez-Calle P., Ceriotti F., Jonker N., Bartlett W.A. (2021). Within- and between-subject biological variation data for tumor markers based on the European Biological Variation Study. Clin. Chem. Lab. Med..

[20] Ceriotti F., Díaz-Garzón Marco J., Fernández-Calle P., Maregnani A., Aarsand A.K., Coskun A., Jonker N., Sandberg S., Carobene A., European Federation of Clinical Chemistry and Laboratory Medicine (EFLM) Working Group on Biological Variation (2020). The European Biological Variation Study (EuBIVAS): Weekly biological variation of cardiac troponin I estimated by the use of two different high-sensitivity cardiac troponin I assays. Clin. Chem. Lab. Med..

[21] Melzi d’Eril G., Tagnochetti T., Nauti A., Klersy C., Papalia A., Vadacca G., Moratti R., Merlini G. (2003). Biological variation of N-terminal pro-brain natriuretic peptide in healthy individuals. Clin. Chem..

[22] Bottani M., Aarsand A.K., Banfi G., Locatelli M., Coşkun A., Díaz-Garzón J., Fernandez-Calle P., Sandberg S., Ceriotti F., Carobene A. (2021). European Biological Variation Study (EuBIVAS): Within- and between-subject biological variation estimates for serum thyroid biomarkers based on weekly samplings from 91 healthy participants. Clin. Chem. Lab. Med..

[23] Wang S., Zhao M., Mu R., Zhang X., Yun K., Shang H. (2018). Biological variation of serum neuron-specific enolase and carbohydrate antigen 724 tumor markers. J. Clin. Lab. Anal..

[24] Zaninotto M., Graziani M.S., Plebani M. (2022). The harmonization issue in laboratory medicine: The commitment of CCLM. Clin. Chem. Lab. Med..

[25] Fraser C.G., Petersen P.H. (1999). Analytical performance characteristics should be judged against objective quality specifications. Clin. Chem..

[26] Carobene A., Marino I., Coşkun A., Serteser M., Unsal I., Guerra E., Bartlett W.A., Sandberg S., Aarsand A.K., Sylte M.S. (2017). The EuBIVAS Project: Within- and Between-Subject Biological Variation Data for Serum Creatinine Using Enzymatic and Alkaline Picrate Methods and Implications for Monitoring. Clin. Chem..

[27] White G.H. (2011). Metrological traceability in clinical biochemistry. Ann. Clin. Biochem..

[28] Nasrollahzadeh D., Aghcheli K., Sotoudeh M., Shakeri R., Persson E.C., Islami F., Kamangar F., Abnet C.C., Boffetta P., Engstrand L. (2011). Accuracy and cut-off values of pepsinogens I, II and gastrin 17 for diagnosis of gastric fundic atrophy: Influence of gastritis. PLoS ONE.

[29] Tong Y., Wang H., Zhao Y., He X., Xu H., Li H., Shuai P., Gong L., Wu H., Xu H. (2021). Serum pepsinogen levels in different regions of China and its influencing factors: A multicenter cross-sectional study. BMC Gastroenterol..

[30] Fu W., Yue Y., Song Y., Zhang S., Shi J., Zhao R., Wang Q., Zhang R. (2024). Comparable analysis of six immunoassays for carcinoembryonic antigen detection. Heliyon.

[31] Walker C., Shuster A., Weber C., Gebhardt K., Scheidegger M., Swartzwelder J., Williams J., Li J. (2021). Comparison of the Siemens Atellica BRAHMS and the Abbott Architect BRAHMS Procalcitonin Assays. Ann. Clin. Lab. Sci..

[32] Dipalo M., Guido L., Micca G., Pittalis S., Locatelli M., Motta A., Bianchi V., Callegari T., Aloe R., Da Rin G. (2015). Multicenter comparison of automated procalcitonin immunoassays. Pract. Lab. Med..

[33] Barr C.E., Funston G., Mounce L.T.A., Pemberton P.W., Howe J.D., Crosbie E.J. (2021). Comparison of two immunoassays for the measurement of serum HE4 for ovarian cancer. Pract. Lab. Med..

[34] Genzen J.R., Burnham C.D., Felder R.A., Hawker C.D., Lippi G., Peck Palmer O.M. (2018). Challenges and Opportunities in Implementing Total Laboratory Automation. Clin. Chem..

[35] Thomson R.B., McElvania E. (2019). Total Laboratory Automation: What Is Gained, What Is Lost, and Who Can Afford It?. Clin. Lab. Med..

